# Endometrial beta3 Integrin profile reflects endometrial receptivity defects in women with unexplained recurrent pregnancy loss

**DOI:** 10.1186/1477-7827-12-53

**Published:** 2014-06-20

**Authors:** Ariane Germeyer, Ricardo F Savaris, Julia Jauckus, Bruce Lessey

**Affiliations:** 1 Department of Gynecological Endocrinology and Reproductive Medicine, University Hospital Heidelberg, Heidelberg, Germany; 2 Departamento de Ginecologia e Obstetrícia, Universidade Federal do Rio Grande do Sul, Porto Alegre, Brazil; 3 Greenville University Medical Center, Greenville, SC, USA

**Keywords:** Beta3 integrin, Recurrent pregnancy loss, Endometrial dating

## Abstract

**Background:**

The pathophysiology of recurrent pregnancy loss (RPL) is still unknown in 50% of the cases. Herein we measure the expression of beta3 integrin subunit, a well-known implantation marker, in women with or without RPL and correlate it with the histological dating of the endometrial tissue.

**Methods:**

LH-timed endometrial biopsies were obtained from cases (RPL; n = 21, age 33.9+/-4.7) and healthy controls (n = 29; age 29.8+/-4.1) during the mid-secretory phase (post ovulatory day: 8 to 10). Endometrial samples were timed histologically according to Noyes’ criteria and underwent immunohistochemical staining for beta3 integrin expression. For statistical analysis the semi-quantitative HSCORE was assessed. Type I (beta3 negative in an out-of-phase endometrium) and Type II defect (beta3 negative in an in-phase endometrium) were also analysed. Statistical analysis was done with Student *t*-test, Mann Whitney *U* test, ANCOVA and *chi* square for trend. Significance was set as *P* < 0.05.

**Results:**

The mean (SD) age in controls was lower compared to cases [(29.8 (4.1) *vs.* 33.9 (4.7) – *P* = 0.001; Student *t*-test)]. The median (range) expression of beta3 integrin in controls and cases was 1.94 (0 to 3.5) *vs.* 0.82 (0 to 3.6), respectively (*P* = 0.001; Mann Whitney *U* test). Significance was still significant after adjusting for age (*P =* 0.03;ANCOVA). The normal positive staining > =0.7 of beta3 integrin subunit and in-phase endometrium was seen in 24 out of 29 (82.8%) controls, but in only 6 out of 21 (28.6%) of cases with RPL; Type I and II defects were seen in 10.3 and 6.9% of controls, while present in 52.4 and 19.1% of cases, respectively (*P* = 0.0005; *chi-*square).

**Conclusions:**

Women with unexplained RPL had significantly reduced integrin expression compared to controls. Our findings underline the need for further molecular analysis of endometrial tissue in affected women.

## Background

Recurrent pregnancy loss (RPL), determined as two or more consecutive abortions by some authors, are seen in 1-3% of couples; an underlying cause, however, is only found in up to 50% [1]. These include embryonic factors, like poor quality embryos with or without karyotype abnormalities, as well as maternal factors, such as uterine malformations, general maternal infections, as well as local inflammation [2], hormonal abnormalities, immunogenic abnormalities (like thyroid antibodies, cardiolipin antibodies or antinuclear antibodies), genetic imbalances and thrombophilic diseases [3].

However, when none of these factors are evident, the recurrent pregnancy losses are classified as idiopathic, because the underlying mechanisms are not well understood. The functional expression of endometrial genes and proteins have been examined, because they compromise the endometrial microenviroment and may therefore contribute to an abnormal foetal-maternal interaction, resulting in pregnancy failure [4]. Furthermore synchronisation between embryonic development and endometrial decidualisation is found to be essential for adequate implantation [5]. Many authors have studied the embryo-endometrium interface in order to elucidate the pregnancy failure [6,7]. Global gene expression analysis [8,9] has determined the adequate endometrial gene and protein expression during the short endometrial receptive stage in the mid-secretory phase, the so-called window of implantation (WOI) [10]. One of these proteins is the αvβ3 integrin [11]. The combined integrin αvβ3 acts as an adhesion promoter via cell-cell interactions and it has been very well characterized within the human endometrium [12]. The αvβ3 integrin is expressed in the glandular epithelium during the window of implantation and translocates into endometrial stroma, if pregnancy occurs [13]. Reduced expression of αvβ3 has been related to unexplained infertility [11] and in women with endometriosis [14]. Xu *et al.*, in a recent publication, did not find any difference in β3 integrin expression in women with RPL compared to normal fertile women [15]. However, these authors analysed the expression of β3 integrin in formalin fixed paraffin embedded (FFPE) samples. It is known that β3 integrin expression in FFPE produces artefacts and should be avoided [16]. In order to find a more accurate expression of β3 integrin subunit in cases with RPL, would be necessary to use frozen sections of endometrial biopsies, instead of FFPE. Other authors reported the expression of avβ3 integrin in endometrial-frozen section and did not find any difference between groups [17,18]. Contrary, other authors found a reduction of αvβ3 integrin during the implantation window in patients with recurrent pregnancy loss, compared to controls, either in frozen sections [19], or in microarray studies [7]. Possible discrepancies among studies could be related to technical differences. Our group has been using the SSA6 antibody to identify the β3 integrin subunit, and the expertise developed in our group to evaluate β3 immunostaining has been validated using NIH software image analysis [20]. This study has two objectives: a) to examine the expression of the αvβ3 integrin in women with RPL compared to healthy controls, b) to compare integrin expression with morphologic dating of the endometrium, which has been widely used to judge the endometrial development and receptivity.

## Methods

### Samples collection and processing

This retrospective case–control study enrolled 21 women with identified unexplained RPL and 29 healthy controls. Demographics are depicted in Table 1. Inclusion criteria of “cases” were 2 or more pregnancy losses in the presence of normal thyroid function, anticardiolipin antibodies, lupus anticoagulant and uterine anatomy. Women with known uterine factors such as fibroids, uterine septa or intrauterine synechia were excluded. Other exclusion criteria were hormonal imbalance (anovulation, polycystic ovary syndrome, diabetes or untreated thyroid disease) and known immunologic, chromosomal or thrombophilic abnormalities. Controls were obtained from regularly cycling, normal fertility proven volunteers, without use of oral contraceptive in the previous 3 months. Endometrial biopsies were obtained from mid-secretory phase, 8–10 days after a urinary LH surge. These biopsies were obtained in sterile conditions, using a pipelle suction curettage at the outpatient gynaecology clinic in Heidelberg, Germany and in Chapel Hill, NC, USA. Endometrial biopsies were divided into 2 portions: one was formalin fixed and paraffin embedded for histological examination with haematoxylin and eosin analysis (for endometrial dating) and the other was frozen in liquid nitrogen for immunohistochemistry assessment of β3 subunit expression. This study was submitted and approved by the Institutional Review Boards of both institutions (Greenville University Medical Center & University of Heidelberg).

**Table 1:** Characteristics of the population

**Characteristics**	**Control n = 29**	**Recurrent pregnancy loss n = 21**	***P *value**
Age (y) mean (SD)	29.8 (4.1)	33.9 (4.7)	0.001^a^
N. of miscarriage mean (SEM)		3.5 (0.3)	
**Endometrial dating**			
in/out-of-phase (n)	26/3	10/11	0.001^b^
**Defect type**^**c **^**n (%)**			
In phase with β3 +	24 (82.8)	6 (28.6)	0.0005^b^
Type I	3 (10.3)	11 (52.4)	
Type II	2 (6.9)	4 (19.1)	

^a^Unpaired Student *t* test.

^b^*Chi*-square for trend.

^c^In phase endometrium has positive β3 integrin subunit.

Type I defect is an out-of-phase endometrium and β3 integrin subunit expression is negative.

Type II defect is an “in-phase” endometrium and β3 integrin subunit expression is negative.

### Immunohistochemical staining

Immunostaining for β3 integrin subunit was performed on endometrial biopsies on 7 μm cryosections using the Vectastain Elite ABC kits (Vector Laboratories, Burlingame, CA) simultaneously in cases and controls in a same batch. Diaminobenzidine (Sigma, St. Louis, MO) was used as chromogen. All procedures were done simultaneously Tissue sections were fixed in 4% paraformaldehyde for 15 minutes. Following a rinse in PBS, pH 7.4, endogenous peroxidase activity was quenched upon incubation for 30 minutes with 0.3% H_2_O_2_ in absolute methanol, followed by a 6-minute rehydration in phosphate buffered saline (PBS). Slides were then incubated with 0.4% Triton-x 100 for 10 minutes. After incubation with blocking serum for 30 minutes at room temperature (4% normal goat serum), sections were incubated with SSA6, a mouse monoclonal antibody against human β3 integrin subunit (1:2000). The specificity of this antibody has been shown and confirmed by other authors [12,21]. Negative controls were analysed on adjacent sections incubated without primary antibody. A PBS rinse was followed by treatment with a secondary antibody consisting of biotinylated goat anti-mouse IgG antibody for 30 minutes (Vector Laboratories). After this incubation, sections were washed and incubated with avidin:biotinylated horseradish peroxidase macromolecular complex for 60 minutes. Diaminobenzidine (DAB) was added and incubated for 8 minutes to complete the reaction and to visualize the immunostaining. As a final step, sections were counterstained with hematoxylin for 5 minutes, dehydrated in a graded series of ethanols, and cleared with xylene. A coverslip was placed over Permount for evaluation by light microscopy. Negative external controls were obtained by withholding the primary antibody and positive internal controls were verified by the endometrial blood vessel staining. The resulting staining was evaluated using a Nikon microscope (Tokyo, Japan), by a single blinded observer (B.A.L.) without knowledge of the subject’s group. Board certified pathologists of both institutions performed the endometrial dating according to Noyes’s criteria [22]. Endometrial dating was considered as out-of-phase, when a discrepancy of 3 days or more was seen between endometrial dating and date of the menstrual cycle.

Assessment of staining intensity and distribution in endometrial glands was made using the semi-quantitative histologic score (HSCORE) system. HSCORE was calculated using the following equation: HSCORE = ∑Pi (i + 1)/100, where i = intensity of staining with a value of 1, 2, or 3, (weak, moderate or strong, respectively) and Pi = the percentage of stained endometrial epithelial cells for each intensity, varying from 0-100%. Low intra-observer (*r* = 0.983; *P* < 0.0001) and inter-observer (*r* = 0.994; *P* < 0.0001) differences for HSCORE in uterine tissues have been previously reported using this technique [23]. The numerical cut-off for a negative result for the αvβ3 integrin was a HSCORE of ≤ 0.7, based on previous ROC analysis [21]. Based on histological dating, in-phase or out-of-phase, and β3 integrin expression, positive/negative, we proposed two types of defects: Type I defect is an out-of-phase endometrium with negative β3 integrin subunit expression, and Type II defect is an “in-phase” endometrium with negative β3 integrin subunit expression [11].

### Statistical analysis

Sample size was calculated using the same parameters as previously published [24]; briefly, it was considered an α and β error of 0.05 and 0.2, respectively; variance of the expression of β3 integrin was considered as 0.79, and a minimal difference of HSCORE between groups was 1.4, i.e., the double of the 0.7 cut-off. These figures yielded, at least, 7 cases per group. Gaussian distribution was confirmed with D'Agostino & Pearson omnibus normality test. If Gaussian distribution was confirmed, unpaired *t*-test was used; otherwise, it was used Mann–Whitney *U* test. ANCOVA was used for adjusting HSCORE between groups, since age in controls and cases (recurrent pregnancy loss) was significantly different. ANCOVA online calculator (http://vassarstats.net/ancova2L.html) and GraphPad Prism 6.0 (GraphPad Software, San Diego, CA) were used for statistical analysis.

## Results

**Figure 1:**
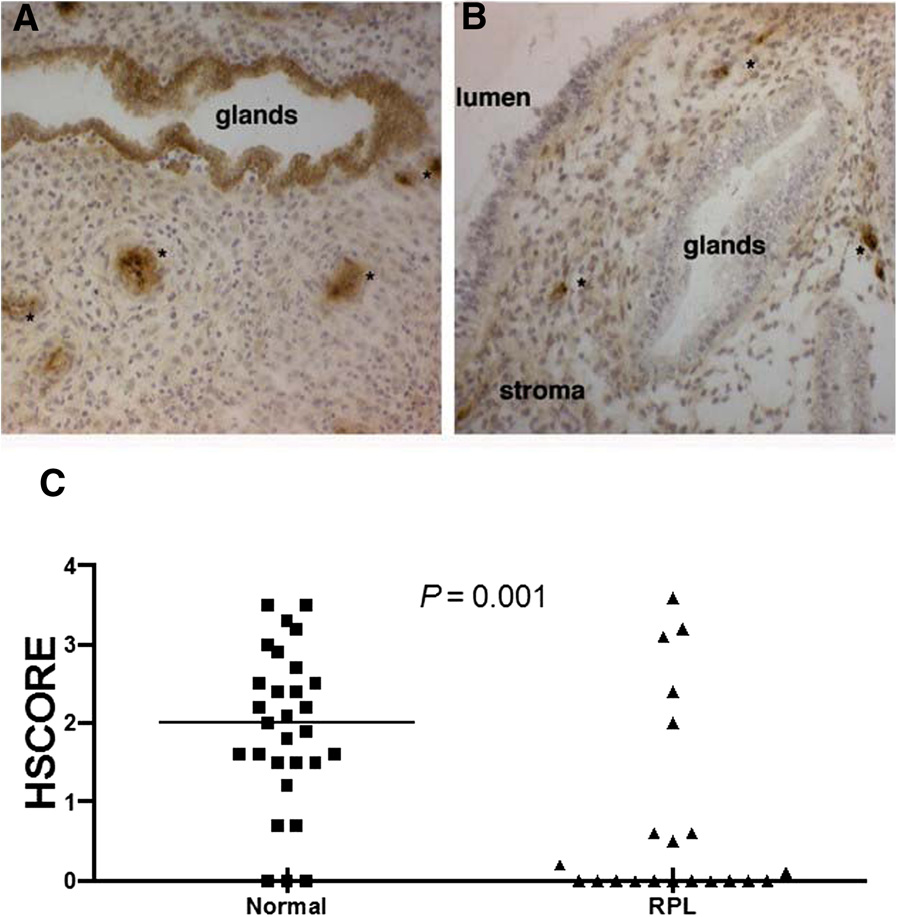
**Immunohistochemistry.** Representative immunohistochemical expression of β3 integrin subunit during the window of implantation in normal fertile women **(A)** and with recurrent pregnancy loss **(B)**. Note the negative expression of integrin in luminal and glandular epithelium in B. Asterisk (*) represents immunostaining for endometrial blood vessel, which is a positive internal control magnification 200x. Individual values are depicted in the graph **(C)** – Unpaired Student *t* test.

Details of the population studied are depicted in Table 1. HSCORE in controls was significantly higher compared to the RPL group (Figure 1). The median (range) expression of β3 in controls and cases was 1.94 (0 to 3.5) *vs.* 0.82 (0 to 3.6), respectively (*P* = 0.001; Mann Whitney *U* test). ANCOVA analysis was conducted to identify if age between groups had influence on β3 subunit integrin expression. Levene’s test of equality of error variances was 0.7. After running ANCOVA analysis, age was adjusted, and the *P* value between groups was 0.03, confirming the significantly difference between groups, despite the age difference between groups.

### Morphological dating and comparison with integrin staining

Compared to controls (n = 29), women with RPL (n = 21) showed more out-of-phase endometrium, i.e., histological delay in maturation. Only 10 out of 21 cases (47.6 - 95 CI: 28.3 to 67.6%) were dated in-phase in RPL samples. In contrast, 26 out 29 controls were in-phase (89.7% - 95 CI: 73.6 to 96.4%). Likewise, positive expression of β3 integrin subunit followed the same pattern in controls. Therefore positive expression of β3 integrin subunit and an in-phase endometrium were present in 24 out of 29 controls (82.8% - 95 CI: 65.5 to 92.4%), while only 6 out of 21 RPL (28.6% -95 CI: 13.8 to 50.0%) samples could be considered “normal”. While a type II defect, determined as in phase endometrium but negative staining for β3 integrin subunit, was found in 6.9% of control samples (CI: 1.9 to 22.0%), 19.1% of RPL samples (CI: 7.7 to 40.0%) were affected (Table 1). The most frequent defect seen in RPL samples (*P* = 0.0005) however was a type I defect with 11 of 21 RPL samples (52.4% - 95 CI: 32.4 to 71.6%) compared to only 3 out of 29 controls (10.3% - 95CI: 3.6 to 26.4%) (Table 1).

## Discussion

The conflicting data on β3 integrin subunit expression in endometrium of women with RPL led us to conduct the present study. Our data indicate that women with unexplained RPL have significantly reduced integrin expression compared to controls. Our results differ from those published from Xu *et al. *[15] and Tuckerman [17]. The probable cause for this discrepancy is the methodology. As it is known, integrin is a membrane adhesion protein and the use of FFPE technique yield artefacts [16], or the variability between observers [17]. We have shown that inexperienced observers in HSCORE tend to give higher HSCOREs [20].

Despite the majority of cases from the RPL group (0.52 – 95 CI: 0.32 to 0.71) demonstrated an out-of-phase endometrium and a negative expression of integrin β3 subunit (Type I defect), an additional 4 cases [0.19 (95 CI:0.07 to 0.4)] presented a type II defect, where integrin expression is negative in an “in-phase” endometrium. These findings demonstrate the need for further molecular analysis, besides the histological assessment of endometrial tissue. This finding is not new. Coutifaris *et al.* suggested that morphological criteria do not differ between fertile and infertile women [25]. Possible causes for this type II defect can be related to the presence of endometriosis [26]. As endometriosis is associated with reduced integrin expression [21], a high-unknown incidence of endometriosis in women with recurrent abortions might be present, and the reduced expression of integrin could be the first clue. This hypothesis is supported by the findings that anti-lamin-1 antibody is reduced in women with endometriosis and with recurrent pregnancy loss [27]. In light of recent evidence linking endometriosis to progesterone resistance [28], these findings support a possible connection between occult or undiagnosed gynaecologic pathology and a loss of normal endometrial receptivity. The fact that integrin expression is reduced in RPL highlights the importance of this adhesion protein for implantation. This reduction is in accordance with other conditions related to infertility. For instance, low integrin expression was seen in women with different degrees of hydrosalpinges [24,29]. After removal of hydrosalpinges, levels of αvb3 integrin increase [30], as well as pregnancies rates [31].

The low expression of integrin in endometrium of women with RPL supports the role of this adhesion molecule in the feto-maternal communication during implantation. In patients with RPL, this reduction of integrin expression may be due to the fact that HOXA10, another implantation marker, regulates the expression of β3 integrin [32]. HOXA10 has been shown to be a modulator of integrin β3, and could be deregulated in endometrium of women with RPL, as suggested by animal studies [33].

There are a few weaknesses in this study. Laparoscopy was not routinely performed in these patients to investigate peritoneal causes such as endometriosis. Likewise, karyotyping was not performed, mainly because it has a low yield for abnormalities (1 to 2%).

The appropriate statistical care, sample size and the appropriate immunohistochemical analysis with a well-established antibody are strengths of this study and corroborate to the accuracy of the results.

## Conclusions

In conclusion, we demonstrate that women with RPL present an endometrial defect during the window of implantation, more specifically, a reduced expression of β3 integrin subunit. Such defects comprised both “in-phase” and “out-of-phase” histology. Further prospective studies are needed to better determine the cause and treatment of these types of endometrial receptivity defects in women with otherwise unexplained RPL. If this correlation remains to be seen, women with unexplained RPL may benefit from additional laparoscopic examination in order to discover potential gynaecological conditions.

## Competing interests

The authors declare that they have no competing interests.

## Authors’ contributions

AG participated in the study design, drafted the manuscript & participated in tissue collection. RFS helped draft the manuscript and performed the statistical analysis. JJ participated in tissue collection and processing. BL conceived of the study, participated in its design and coordination, participated in tissue collection, reviewed the manuscript & performed the Immunohistochemistry staining and analysis. All authors read and approved the final manuscript.
